# Unified selective sorting approach to analyse multi-electrode extracellular data

**DOI:** 10.1038/srep28533

**Published:** 2016-06-24

**Authors:** R. Veerabhadrappa, C. P. Lim, T. T. Nguyen, M. Berk, S. J. Tye, P. Monaghan, S. Nahavandi, A. Bhatti

**Affiliations:** 1 Institute for Intelligent Systems Research and Innovation, Deakin University, Vic 3216, Australia; 2 IMPACT Strategic Research Centre, Barwon Health, Deakin University, Vic 3216, Australia; 3 Department of Psychiatry & Psychology, Mayo Clinic, Rochester, MN 55905, USA; 4 Australian Animal Health Laboratory, CSIRO, Vic 3219, Australia

## Abstract

Extracellular data analysis has become a quintessential method for understanding the neurophysiological responses to stimuli. This demands stringent techniques owing to the complicated nature of the recording environment. In this paper, we highlight the challenges in extracellular multi-electrode recording and data analysis as well as the limitations pertaining to some of the currently employed methodologies. To address some of the challenges, we present a unified algorithm in the form of selective sorting. Selective sorting is modelled around hypothesized generative model, which addresses the natural phenomena of spikes triggered by an intricate neuronal population. The algorithm incorporates Cepstrum of Bispectrum, ad hoc clustering algorithms, wavelet transforms, least square and correlation concepts which strategically tailors a sequence to characterize and form distinctive clusters. Additionally, we demonstrate the influence of noise modelled wavelets to sort overlapping spikes. The algorithm is evaluated using both raw and synthesized data sets with different levels of complexity and the performances are tabulated for comparison using widely accepted qualitative and quantitative indicators.

Neurophysiological studies are of paramount importance in revealing the underlying behaviours and properties of neurons and eventually providing a good understanding of the human nervous system. The studies have proved to be very important in the development of neuro-prosthetics and Brain Machine Interface (BMI) devices. As an example, intra-neuronal recordings from the primary motor cortex have been investigated to develop neural decoders that can eventually drive artificial prostheses or machines^1^. Further, the contribution of these studies in understanding neurological disorders are extremely valued, especially, the use of intracranial electrodes to gather information pertaining to epileptic patients^2^. Indeed, MEA’s have been employed to understand the influence of gamma-protocadherine, which regulates the endurance of a neural network and the generation of new synapses^3^.

One of the key aspects of neurophysiological studies involves the tapping of intra-neuronal signals, so as to decipher the neural networks collective behaviours without disrupting their natural functioning. Extracellular recordings are the preferred techniques to aid in neurophysiological studies, and the recordings can be mainly grouped into two categories, that is: *in-vivo* (invasive) and *in-vitro* (non-invasive). *In-vivo* recording techniques use a micro-electrode like probe or a tetrode (probe with four electrodes) to be surgically implanted onto a region of observation, in which intra-neuronal activities are recorded^4^. In contrast, *in-vitro* recording techniques use a micro-electrode array (MEA) with the cell samples cultured in a petri dish^5^. Similarly, active cell specimens from animals are collected and placed on the micro-electrodes from which the intra-neuronal activities are recorded^6^.

## Problem Statement

Irrespective of the recording techniques used, the intricate nature of the nervous system poses major problems during tapping and processing of intra-neuronal signals. The main attribute of any intra-neuronal activity is the pattern made up of action potential followed by a refractory period, which is referred to as a neuronal spike^7^,^8^. A major problem associated with the processing of any intra-neuronal recording is that each electrode is subjected to more than one neuronal activity at any instance^9^. The electrode closer to a neuron renders stronger signals to be picked up by the channel, whilst action potential from neighbouring neurons superimposes upon the stronger ones contributing as noise to the channel. Additionally, noise may also be contributed by the recording unit, processing system and the surrounding environment^10^ resulting in uncharacteristic spike events. To interpret the collective behaviour it becomes imperative to distinguish each neurons activity both in time and space under reduced influence of noise.

## Existing Procedures and Drawbacks

The process of identifying the number of neurons and the spike times associated with each neuron is referred to as spike sorting. The major challenges in our sequential spike sorting algorithm can be broadly summarized as follows^4^,^9^.

- Detecting the instances at which the spiking activity appears in each voltage channel of the recording device;
- Extracting the spike shapes for clustering; and
- Estimating overlapping spikes.

Each of the aforementioned steps depend on the results of its preceding steps. The precision of results at each step is of utmost importance as any error accumulates through every step, thus degrading the performance of the algorithm. Simple spike detection techniques employ a hard thresholding, which is a straightforward technique. Each channel voltage is examined and through visualisation a statistical estimator such as: standard deviation of the channel signal^8^,^11^, root mean square^4^,^12^ or standard deviation of the background noise^13^,^14^ is employed to identify a spike event. Generally, the performance of these simple techniques degrades under low Signal-to-Noise Ratio (SNR)^15^. A more comprehensive method is described in ref. 16 where a window-based spike detector is proposed. Specifically, a preset window of defined duration scans for a positive peak followed by negative peak and corresponds the span to be a spike waveform. Nevertheless, the extracted spike duration represents only one spike and any secondary positive peaks will be ignored. Overlapped spike shapes with their unrepresentative appearance, as compared with typical spike shapes are considered as noisy or distorted waveforms.

Assuming that the spikes were detected comprehensively, appropriate feature vectors are required to be identified prior to clustering. Initially, spike shapes are extracted using windowed discriminators, as discussed in the previous paragraph. And to highlight the features of interest, the extracted spikes are subjected to a suitable transformation. Principle component analysis has been widely adopted in selecting the prominent features^4^,^17^. Alternatively, a random number of features that display higher deviation from normality, following a normality test, are chosen as the inputs for clustering^13^,^18^. It has been observed that the deviation results are dependent on the transformation method used on the data set and no standard is defined to test the quality of the selected features. Besides that, a poor data transformation could result in poor selection of features and could hinder the performance of clustering.

Standard clustering algorithms including k-means, partition around medoids (PAM) and hierarchical clustering require prior knowledge of number of clusters, that is the K value^19^. The lack of ground truth forces one to use brute-force experiments to approximate the K-value. This dilemma of choosing an optimal K value leaves the general clustering algorithms not well disposed to sort neuronal spikes. Besides, many shortcomings of k-means are described in ref. 20, making it a even less favourable spike sorting method. To overcome these shortcomings, many novel clustering algorithms have been proposed, such as Wave_clus, which is an open-source spike sorting program that incorporates the super paramagnetic clustering (SPC) algorithm^13^,^21^. Klustakwik is another open-source software based on genome clustering and CEM which uses the idea of classification by expectation maximization^4^. The Ordering Points To Identify Clustering Structure (OPTICS) is another algorithm^22^ developed to compensate the complex feature selection processes^8^. Despite the availability of such power clustering procedures, they are often vulnerable under overlapping spike shapes.

Traditional approach employ Mclust, a manual procedure of forming clusters. Further, the clusters are visually examined and a clear un-contaminated spike shape is identified to represent the subset of a spike waveforms^11^. The representative spike waveforms were subtracted from an overlapping event and the one which resulted in the least channel voltage error was considered as the best match. This manual clustering and the arbitrary matching procedure based on spike waveforms voltage distribution across a channel, would not yield a good result under complex neuron populations and long recording durations. This shortcoming is addressed in ref. 23, where the background noise covariance is used to enhance the principal spike component in a channel, by targeting some specific spikes shapes. The model proposed in ref. 8 is based on the maximum likelihood estimation incorporating noise covariance characterization into their matching process. However, the algorithms lacks a definitive generative model, does not address any previous ground truth estimation processes and, as such the model is less attractive.

A more sophisticated model is proposed in refs 24 and 25, which assumes the channel voltage to be the result of a convolution between impulse spike train and spike shapes. The process also considers background noise distinction and greedy matching procedures to segregate overlapping spikes. The basic notion of the greedy matching procedure simply identifies peaks as spike events and tends to recognize any detected events into a predefined groups formed by clustering algorithms. The lack of a comprehensive thresholding technique means that the greedy matching procedure identifies far too many false positives.

One common drawback of all the aforementioned models is that they fail to rationally address the spike event detection. Therefore, when the channel SNR is low these models perform ineffectively.

## Proposed Approach and Improvements

The proposed selective sorting model addresses the problem of spike detection in novel way. We have adapted the concept of Cepstrum of Bispectrum (CoB) based spike detection owing to its effective results even at a low SNR^26^. Window-based spike waveform extractors are used to extract spike shapes in the detected region^16^. Instances at which spikes are detected are stored as index information, which is later used during statistical estimation. The spikes are subjected to wavelet transformation and a test of normality is employed to choose the best features for clustering^18^.

A novel probability density function (pdf) based technique is introduced to visually examine the quality of the chosen features. The chosen features are subjected to three different clustering procedures including SPC^21^, Klustakwik^25^ and OPTICS^22^,^27^,^28^. The results are statistically compared, and the one with a better overall score is chosen for estimation of overlapping spikes. The statistical estimation introduced here incorporates the principal spike shape employing linear regression, noise distinction based filtration technique, which is followed by matching and iterative elimination through a model formulated in refs 8 and 23. The advantage of this algorithm is, instead of greedily choosing the spikes with the maximum peak^8^,^24^,^25^ the algorithm is restricted to only those indices detected during spike detection.

## Selective Sorting Algorithm

**Figure 1:**
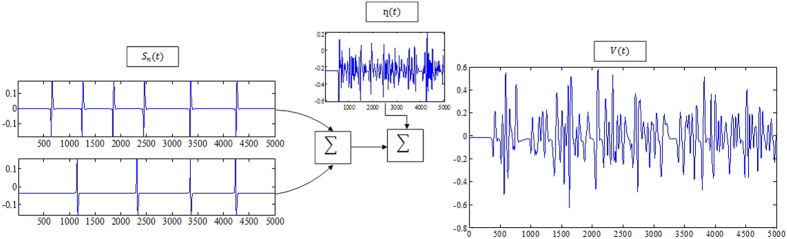
Summing of spike trains with noise giving the final extracellular voltage *v*(*t*).

**Figure 2:**
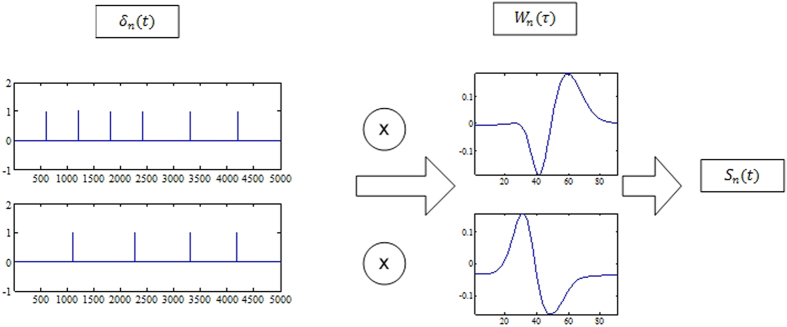
Impulse responses of individual neurons and their respective spike waveforms leading to their respective spike trains *s*_*n*_.

**Figure 3:**
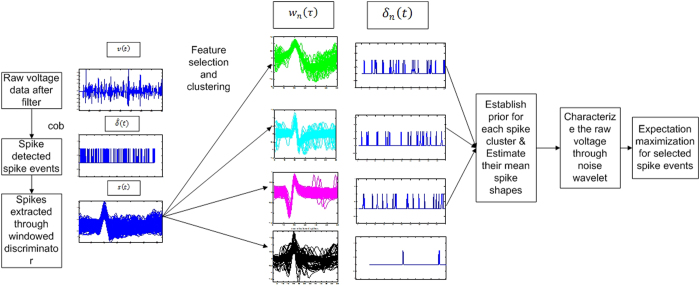
Overview of the proposed Selective sorting algorithm.

Figures 1 and 2 summarizes the Smith and Mtetwa’s^29^ model for generating extracellular signals which is used as a basis for tailoring the proposed algorithm. An overview of the selective spike sorting algorithm is described in Fig. 3. Accordingly, the voltage information *v*(*t*) on a single channel can be formulated using (1) as


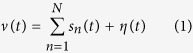


where *n* is the number of neurons assumed to be spiking over discrete time *t, N* is total number of neurons under consideration, *s*_*n*_ is the spike train of the *n*th neuron and *η*(*t*) is noise. Spike train *s*_*n*_ can be realized as representing the voltage information for any *n*th neuron without system noise *η* as


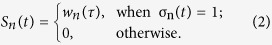


where *w*_*n*_(*τ*) is a distinct spike waveform associated with the *n*th neuron, *τ* is the length of each spike waveform, which is approximately 2.5 *ms* or 60 samples at 24 kHz sampling rate. The proposed algorithm carefully analyses the synthesizing process described by 1 and 2. At every step, we aim to distinguish the input impulse sequence *δ*_*n*_(*t*) from the extracellular voltage *ν*(*t*). To facilitate this process establishing logical ground truths is very important, which can be conveyed by an approximate estimate of the number of neurons and their spiking events *δ*_*n*_(*t*).

With ground truth estimation as our foremost motivation and, considering the fact that *ν*(*t*) is made up of a combination of spike train *s*_*n*_(*t*) from *n* neurons and system noise *η*(*t*), as described in 1. We examine the spike sorting process by assuming an ideal case i.e. in a noise-free environment where only a single neuron is actively spiking during the recording process. As such, for *n* = 1 and *η*(*t*) = 0 in 1, *ν*(*t*) = *s*_*n*_(*t*) = *s*(*t*) and 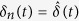. Extending this method for real case scenarios, where more than one neurons have contributed to the channel voltage, 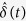 represents a combined input sequence, i.e. if *n* ≥ 1 then 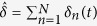.

Given 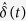, one simple method to segregate the combined sequence into *n* neuronal sequence *δ*_*n*_(*t*) is to identify all *M* spike shapes 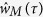 and group the similar ones based on a clustering procedure. It should be noted that not all spike shapes can be grouped as they include many overlapped spike shapes. The defined groups are used as a base to approximate the *n* value that is *δ*_*n*_(*t*) from 2. This is under the assumption that 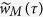 represents the mean or an average spike shape for each group similar to *w*_*n*_. This leads to the probability of finding any *n*th spike waveform *w*_*n*_ given 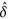 which follows a simple likelihood principle as


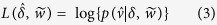


For a valid 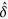 the chances of finding the *n*th neuron follow the Bernoulli’s principle as


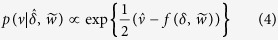


where *f* refers to the transfer function defined by 2. By estimating the likelihood of all *n* neurons at all 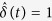, the one with the highest probability constitutes the ideal choice, which reflects on the respective *δ*_*n*_(*t*) accordingly.

## Methods

We summarize the concept of CoB-based spike detection from^26^. The CoB technique assumes a model where the channel voltage *ν*(*t*) is the resultant of a binomial incoming process *δ*_*n*_(*t*) filtered through *f*(*t*), filter transfer function made up of the intra-neuronal spike shape *w*_*n*_(*τ*) and the spike transfer characteristic defined in 2, summed with noise *η*(*t*). By filtering *ν*(*t*) through an inverse filter *f*^−1^(*t*), the input sequence can be recovered along with its noisy component.

### Estimating the combined impulse sequence

If *f*(*t*) represents the filter transfer function in the time domain whereas *F*(*n*) in the frequency domain obtained by taking the Fourier transform of *f*(*t*). Bispectrum *B*_*ν*_(*n, m*) of voltage *ν*(*t*) is estimated by taking the third moment of 2-dimensional Fourier transformation at frequency components *n* and *m* using 5 as


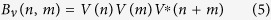


Ceptrum can be computed by taking an inverse Fourier transform in logarithm of 5 at frequency *m* as


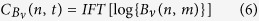


or by assuming *V* to be result of 2 in which case 6 can be rewritten as


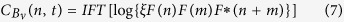


where *ξ* represents the skewness of *δ*(*t*). The term *F*(*n*) can be computed by solving 7 for log as


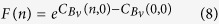


Now *f*^−1^(*t*) can be computed by taking the inverse Fourier transform of *F*^−1^(*n*). To recover the input sequence *δ*_*n*_(*t*) from its noise term, the filtered signal is further subjected to a stationary discrete wavelet transform using the coiflet wavelet *δ*_*n*_(*t*) owing to the fact that CoB just identifies the spike times but does not segregate it to their respective neurons.

**Figure 4:**
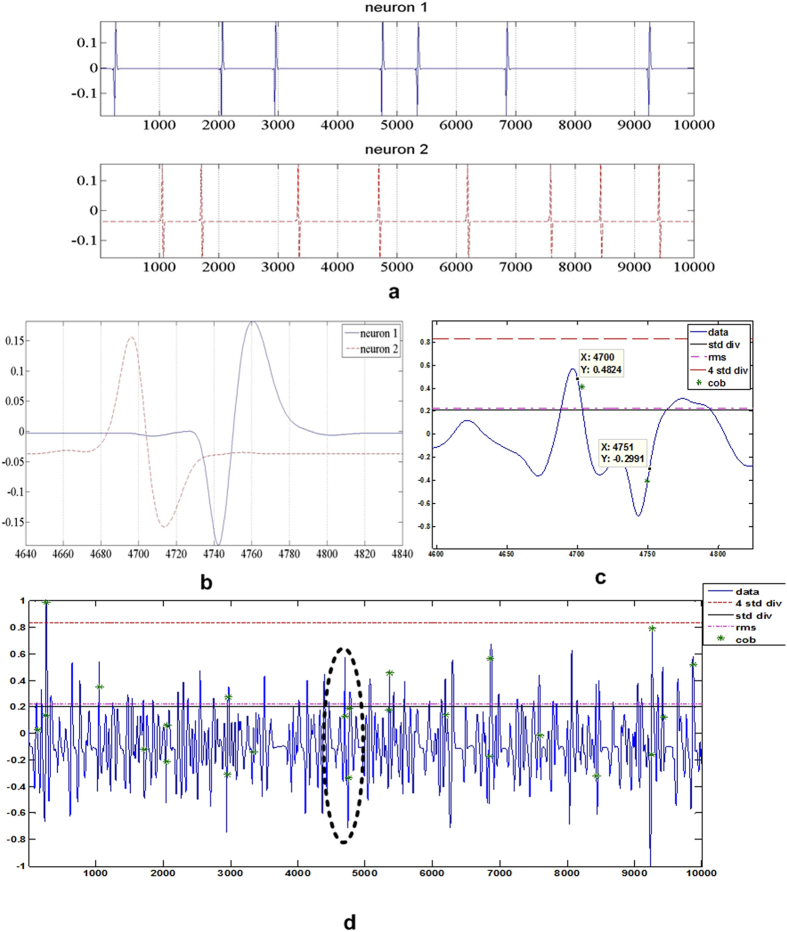
(**a**) Individual input spike train from each neuron which is summed to produce channel data, (**b**) Magnified part from (**a**), clearly showing the overlap interval, (**c**). The two green stars are the intervals of peaks detected by CoB, (**d**). Channel voltage after summing the spike trains in (**a**) which is further correlated by spikes originating from neighbouring neurons and very low Gaussian noise. The green stars indicate the spikes detected after applying CoB method.

As an example, we synthesize a single channel voltage data *v*(*t*) using the model described in ref. 29. For each of the distribution pattern shown in Fig. 4(a) our synthesizing model uses two distinct spike shapes displayed in Fig. 4(b). The model establishes the stringent case of overlapping and the capability of CoB to identify two overlapping spike events with a near synchronous overlap as demonstrated in Fig. 4(c). The resulting voltage *v*(*t*) resembles as shown in Fig. 4(d).

### Establishing and approximating the ground truths

From 2, the recovered sequence 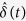 is modelled such that the spiked instance *t* is set to 1 as presence and 0 as absence. The following procedure relays the methodology to distinguish this sequence into their respective neurons *δ*_*n*_(*t*).

#### Spike waveforms extraction

**Figure 5:**
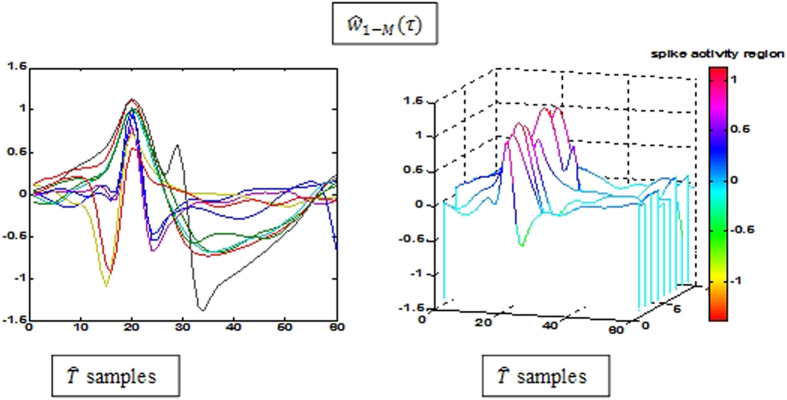
Spike extraction and aligning.

The windowed discriminator technique similar to the one described in ref. 16 is used to extract spike waveforms. For each instance of 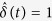 a waveform 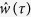 of approximately 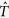 samples are extracted where *τ* ranges between 1 and 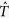. Secondary spike found within 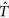 are neglected and only the maximum peak for each waveform is considered. For uniformity and simplification of the feature selection procedure the waveforms are organised with all their peaks lined up as shown in Fig. 5.

#### Construction of feature set

Feature selection follows the same procedure as described in ref. 13, where all *M* number of extracted spike waveforms, 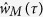, are decomposed using Haar wavelets. The feature set is constructed by identifying 10 best features, which have a better deviation from normality. It is also possible that while some features are identified with better results in KS-test, they do not favour clustering. Therefore, each identified feature set is cross verified by plotting against their respective pdfs using the following expression


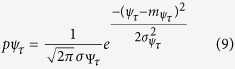


where, *ψ*_*τ*_ represents the wavelet transform at any *τ* over the dimension M, while 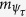 and 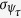 are the respective standard deviation.

#### Clustering

Three major clustering algorithms specially designed to enhance spike sorting are described. It is worth pointing out that real spike data always differ from synthetic spike data in terms of SNR, ground truths, and their unknown statistical distribution. The features selected in the previous step are subject to all the following clustering algorithms to better approximate the ground truths.

##### Super-paramagnetic clustering

Super-paramagnetic clustering (SPC) is devised around the concept of the ising Model^27^,^30^ as in a chemical bonding of any lattice structure. Instead of restricting the number of states to just two (+ or −) q-states are introduced as in the potts model^31^. Each of the *M* waveforms is initially assigned to one of the q-states. The Euclidean distance *e*_*i*,*j*_ of all *M* spikes are estimated and the shortest path is derived by assuming that the neighbours are formed within a specified *K* value as


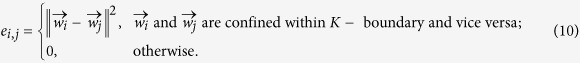


where *i, j* = 1, 2, ··· *M*. The interaction strength *J*_*i*,*j*_ is evaluated as


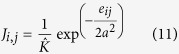


where 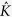 is the average neighbours identified for each *i* and *j* and *a* is the average of *e*_*ij*_^27^. The main feature of SPC is that by varying temperature *T* from low to high value over several Monte Carlo simulations, the system undergoes magnetisation changes traversing from the ferromagnetic state to the paramagnetic state. The states simultaneously flip and take up a different q–value and the new states are defined at the super-paramagnetic phase forming the required clusters^30^,^31^. The probability of two neighbouring features sets change their states *s*_*i*_ and *s*_*j*_ to a new state, which is determined by the thermal average of point-to-point correlation function 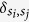 as is defined in refs 12 and 13. This probability is expressed as


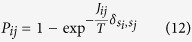


##### Klustakwik

Klustakwik has been an integral part of the spike sorting algorithm proposed in refs 12 and 32. The CEM algorithm by Celeux and Govaert^33^ is used as a platform to construct Klustakwik *ver*–1.5 an unsupervised clustering algorithm. The Expectation Maximisation method is adopted to estimate the maximum likelihood by incorporating classification between the expectation and maximisation method.

Klustakwik partitions a feature set into *K* partitions by iteratively splitting a defined cluster or deleting and re-assigning points from a cluster and simultaneously monitoring whether any of the actions improve the performance. Many versions of Klustakwik have been developed with its predecessor constructed on the genome sequence clustering (gclust) platform and are publicly accessible^4^,^28^.

##### OPTICS

The OPTICS algorithm was developed to abstain from using the tedious feature selection processes^8^. This algorithm partitions the data solely on the basis of set theory and Euclidean distance between the waveforms *w*_*n*_(*τ*). A pre-specified number of the minimum data samples *K* to be present in any group is used to estimate the boundary. The border samples which do not comprehensively satisfy the boundary conditions are not grouped^22^.

### Statistical estimation

From 3 to estimate the likelihood, we need to establish a subject which clearly distinguishes noise from the real data by isolating the overall noise characteristics from the clustered information. Putative spike waveforms 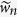 provide a platform to extract the noise attributes and by filtering *ν*(*t*) through a transfer function modelled using the covariance of noise. It is possible to enhance the components of 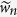 in *ν*. The likelihood *L*_*n*_ is computed for each *n* neuron. Following the Bernoulli’s principle an *n*^*th*^ *L* with better probability is considered as a valid match and its respective sequence *δ*_*n*_(*t*) is set to 1 to acknowledge the presence of spike. The statistical estimation steps for sorting overlapped spikes can be summarized as

- Estimating putative spike waveforms 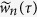.
- Estimating noise *ν*_Δ_(*t*) and its covariance.
- Computing the coiflet type filter transfer function and filtering the channel voltage 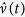.
- Identifying the non-clustered index from 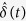 and performing a likelihood estimation for the probability of finding the *n*th waveform and updating the respective input sequence *δ*_*n*_(*t*).

#### Estimating putative spike waveforms

From 2, it can be assumed that a spike train *s*_*n*_(*t*) is associated with a distinct spike shape *w*_*n*_(*τ*) representing an important characteristic of a neuron. Therefore, the putative spike waveforms 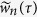 estimated by taking an average of spike shapes from each cluster approximate closely to the distinct spike shape. The clustering information is used to calculate spike impulse response *δ*_*n*_(*t*) of each neuron as


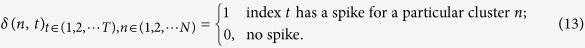


Each cluster is assumed to be originating from a particular neuron. As such, the indices of clustered spike waveforms are set to 1 and the rest to 0 over a complete *t* axis. The least squares linear regression method^24^ is adopted to estimate the putative spike shapes 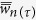 as presented in 13. This is achieved to accommodate the transfer function described in 2 as opposed to the convolution model.


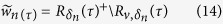


where 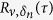 is the cross correlation between impulse response *δ*_*n*(*τ*)_ and the channel voltage *ν*(*t*) for any *n*th neuron, 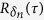 is the auto-correlation of *δ*_*n*_(*τ*), + indicates the toeplitz matrix and\is the pseudo-inverse function. The indices detected correspond to the peak of the spike waveforms action potential and not the start of the spike waveform. Therefore the length of spike waveform *τ* has to be carefully adjusted between −ve and +ve lags such that the peak is at the 0.

#### Noise covariance estimation

All the unwanted data from the channel voltage *ν*(*t*) are considered as noise. This includes system noise *η*(*t*) and the unclustered spike waveforms. A simple way to extract noise is to target those clustered indices and eliminate their spike shapes 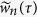 from *ν*(*t*). The residual *ν*_Δ_(*t*) contains noise, the unclustered spike waveforms along with difference between 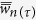 and 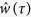 at each clustered index. Auto covariance 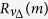 of the residual noise *ν*_Δ_ represents the noise covariance of any particular channel as


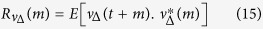


#### Estimating coiflet type filter transfer function

**Figure 6:**
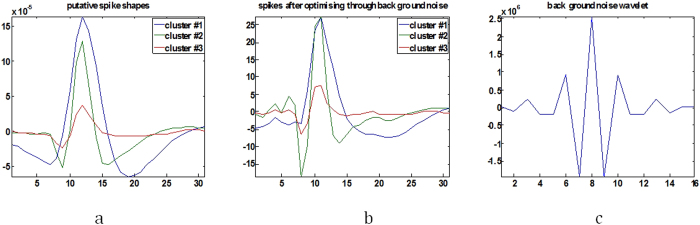
(**a**) Putative spike shapes from original data, (**b**) Spike shapes recovered after noise optimization (**c**) Noise modeled wavelet.

Coiflet type wavelets are generally used to compute wavelet transform^34^,^35^ of a signal which can enhance the signal strength over its noise component. By filtering the channel voltage *ν*(*t*) through the coiflet filter constructed using noise covariance 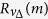 of a finite length *m*, it is possible to accentuate the presence of putative spike shape features. The Toeplitz matrix of 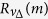 is initially computed and the centre column from the matrix defines the coiflet transfer function *f*(*m*). Figure 6(b) shows the spike shapes extracted from filtered 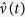 and Fig. 6(c) displays the estimated wavelet resembling noise characteristic filter. The filtering process has an overall effect on *ν*(*t*) therefore the putative spike shapes are re-estimated to produce 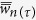.

#### Prediction and elimination

With the new putative spike shapes 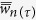, filtered voltage 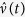 and target instances 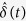, the maximum likelihood estimation described in 3 can be re-written to accommodate for *L*_*n*_, i.e. the likelihood estimated for each *n* neurons as


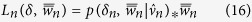


*L*_*n*_ maximises the chances of finding 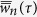 from the filtered voltage 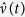 at any *t*th instance of 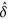.

Note that *δ*_*n*_ described in 1 and 2 follows the Bernoullis principle where at any *t* the presence or absence of 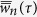 is indicated by *δ*_*n*_(*t*) equal to 1 or 0. With this assumption 16 becomes


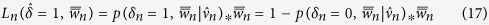


Assuming the condition for *δ*_*n*_ = 1 and substituting 4 into 17 and considering the log likelihood, we obtain


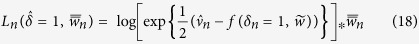



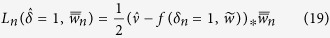


Neglecting the scaling factor of 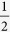 in 19 we have


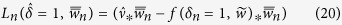


It should be noted that the entire operation is performed only for all 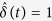 and assuming that *δ*_*n*_ = 1 for each *n* neurons. According to the Bernoulli principle, the matching process follows a Bayesian distribution according to which there is either a chance of 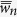 being present at that instance or not. At any 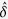 the maximum of the *n*th *L* wins the prediction and its respective *δ*_*n*_(*t*) is set to 1.

## Data

Two categories of data sets are used to test the performance of the proposed system. The data sets were chosen such that the degree of complexity could be augmented upon successful and promising results.

### Synthetic data

A synthetic data set C_Difficult1_noise(xx) (abbreviated as D1n-(XX)) from^36^ was used to test various phases of our algorithm. The ubiquitous nature of the availability of this data set with ground truth information makes it a popular choice for performance evaluation and comparison. Note that “XX” corresponds to the noise levels relative to the amplitude of the spike classes. Four data sets with noise levels 0.05, 0.1, 0.15 and 0.2 were employed for comparative performance analysis. The data set is constructed with three distinct spike shapes sampled at 24 kHz with a known number of neurons (3 in this case) and overlapped spikes.

### Extracellular data

It is observed that *in-vivo* recordings are not stable; therefore *in-vitro* recordings are preferred in testing and development of any such algorithms^37^. Henceforth, we chose to evaluate the algorithm using publicly accessible data sets^36^ as well as multi-electrode raw data from an amphibian retina^6^,^38^. The eye of an amphibian animal is enucleated and highly precision surgical equipments are used to isolate the lens of the eye and the cornea from its posterior half. The eyecup is now filtered to extract the retina specimen form the surrounding vitreous. And finally, through careful dissection the pigment epithelium is removed from the eyecup resulting in a retina specimen of approximately 1.5mm radius. The dissection was performed in ringer’s solution which will be transferred along with the extracted specimen on to the electrode array recording bin.

**Figure 7:**
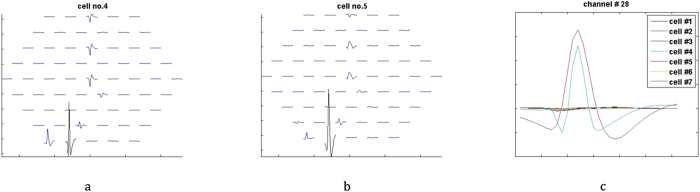
Spikes across 61-electrode MEA. (**a,b**) Show the spike shape of cell-4 and cell-5 scattered across all 61 electrodes. (**c**) Displays the spike shapes of all the cells at 28th channel and the two peaks indicate the cell-4 in cyan and cell-5 in magenta while the rest of the cells does not show much activity on this channel.

The recordings were performed by Meister *et al*.^6^,^38^ using a dense electrode array comprising of 61 electrodes. The electrode array was fabricated by Regehr *et al*.^39^, designed by Pine and Gilbert^40^ and the development was supported by Caltech and Stanford Center for Integrated Systems. The dimension of each electrode was approximately 5 *μm* radius and the spacing between the electrodes was 70 *μm*. Figure 7 is an overview of arrangement of electrodes in the MEA. During the recording process, the isolated cells are kept alive through an oxygenated solution and the retina cells are subjected to different coloured frames projected using an RGB display monitor. The electrical activity due to the stimuli response for each frame is recorded across an MEA comprising of 61 electrodes^6^,^38^,^40^.

To establish the data set, we examine the spiking activity of 7 cells across all 61 electrodes, the recording comprises of 1.5 million samples. To narrow the importance of our algorithm on superseding spikes we observe the activity of cells 4 and 5 as shown in Fig. 7. The traces of spikes for cells 4 and 5 have equal chances of being found on a certain channel indicated by spikes with the highest amplitude. Therefore, this channel (identified as the 28th channel) presents sufficient chances of spike shapes from either cell to supersede one another.

## Results and Performance Evaluation

The algorithm is examined through both quantitative and qualitative analyses and the results are compared to evaluate the performance of the algorithm. The synthetic data sets are the preferred choice for preliminary evaluation due to the availability of the ground truth information required to step-wise testing and calibration of the algorithm. The general quantitative analyses mentioned in refs 8, 25 and 26 include calculating the number of true spikes, number of false spikes, number of neurons approximated, number of correctly identified overlapped spikes and number of correctly sorted overlapped spikes. Qualitative analysis is the best way to compare the recovered spike shapes and spike trains especially with the raw data when there is inadequate information. The raw data relies on approximated generative information at every stage of the analysis procedure. Albeit, the raw data set used in this context provides partial generative information and initial data analysis results. We have compared our results with the original results which show substantial and effective improvement over its original predecessor.

**Figure 8:**
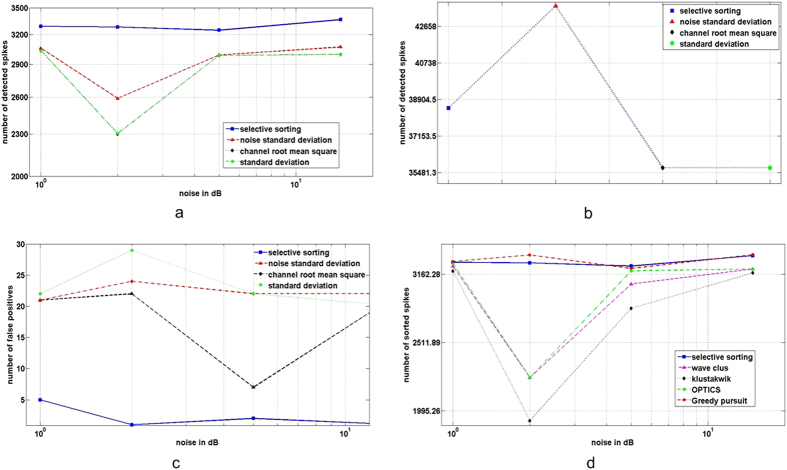
Performance comparison of selective sorting with its counterparts, (**a**) Comparison of spike detection algorithms for sample data set C-Difficult1-noise^37^, (**b**) Comparison of spike detection algorithm for raw dataset^6^,^33^, (**c**) Comparison of false positive estimates for each of the existing detection algorithms and (**d**) Comparison of sorting algorithms.

The results of spikes detected by selective sorting is compared with a number of popularly adopted techniques including noise standard deviation, root mean square of channel voltage and standard deviation of the channel^4^,^8^,^12^,^13^,^14^. Figure 8(a) demonstrates the superiority of selective sorting to identify a larger number of spikes. Figure 8(c) shows the number of falsely identified spike events, which validates the accuracy of selective sorting to correctly identify spike events. To maintain uniformity Fig. 8(a,c) are constructed using the same data set. Figure 8(b) shows the number of spike events detected for the raw extracellular data set. Although, the noise standard deviation displays an improvement in the number of spikes detected, the higher false positive readings in Fig. 8(c) reduces the performance of the technique, making it less favourable. Additionally, Fig. 8(c) is accountable owing to its known ground truth information, which is not available for the raw data set.

**Figure 9:**
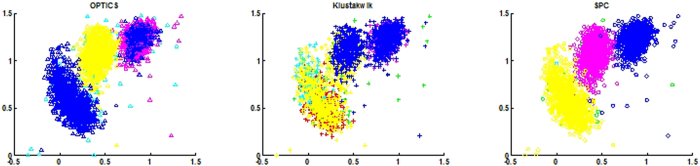
Visual comparison of clustering procedure outcomes for a sample data set.

Table 1 demonstrates a summary of results obtained by various ad hoc clustering approaches and the clustering procedure employed in selective sorting, along with the original information used to simulate the data set. Ideally, the spike sorting algorithms should aim to achieve the results similar to the ground truth information. The results in Table 1 compare the number of spikes allocated into any cluster and the number of clusters formed by any clustering approach for a particular data sets. For example, for the data set *D*1*n* − 0.1, Wave_clus produces 5 clusters with 639, 470, 653, 424 *and*, 1059 spikes respectively and, the * indicates an over-lap spike cluster. The results for Wave_clus and Klustakwik were generated using the online portal^41^, which uses features selection via the KS-test. The result for OPTICS were generated using the conventional method which uses no feature selection processes^8^. It is evident from the results in Table 1 and Fig. 9 that the clustering process employed in selective spike sorting is very effective in identifying appropriate features, defining clusters corresponding to the right number of neurons and displays better sensitivity to overlapping spikes.

The greedy pursuit algorithms: Continuous basis pursuit (CBP), binary pursuit and Bayesian algorithm based greedy algorithms fairly share the same basic principle in their methodology, albeit, the results vary marginally depending on the algorithm used. For comparison purposes, we implemented the greedy pursuit method as described in ref. 25. Figure 8(d) highlights the final results in terms of number of spikes after overall computation of selective spike sorting, clustering algorithms and greedy pursuit. The superior advantage offered by greedy pursuit and selective spike sorting to sort overlapping spikes over other clustering algorithms could be visualised from the graph in Fig. 8(d). Further, it can be inferred from the results summarized in Table 2 that, the overall performance of selective sorting is very effective owing to its impressive number of sorted spikes to number of false positive’s ratio and rightfully targeting the genuine spikes as compared to greedy pursuit.

**Figure 10:**
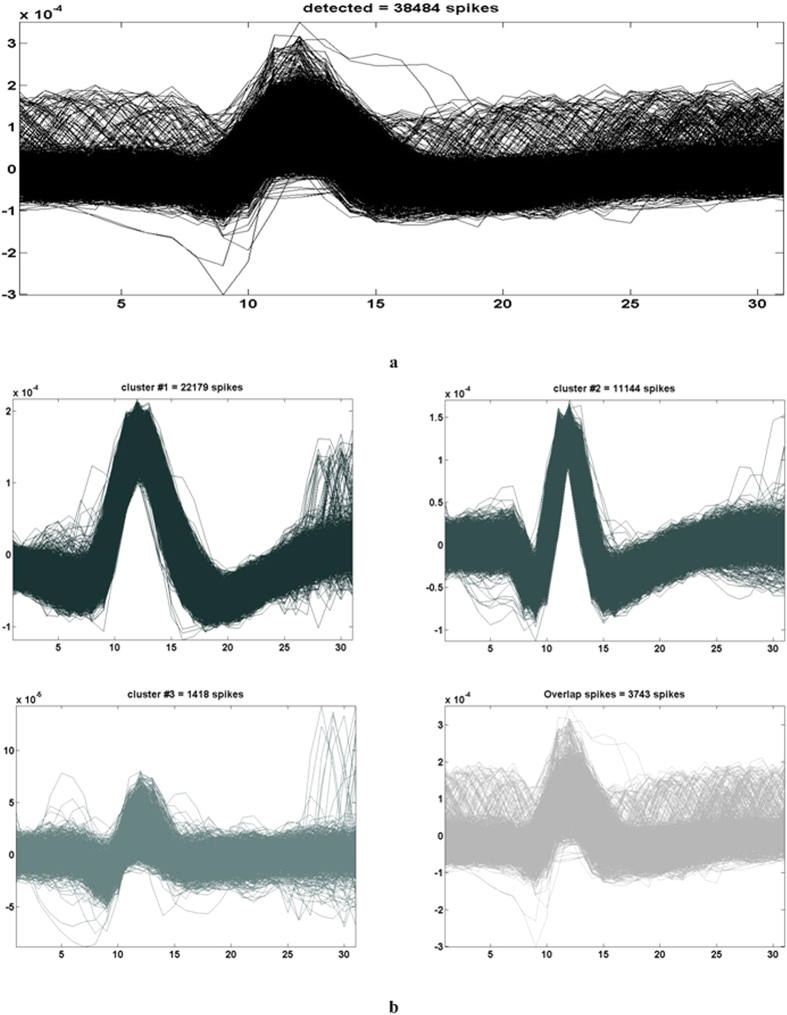
Results for the raw data^6^,^33^ with selective sorting (**a**) Spikes detected using CoB and extracted using a windowed discriminator, (**b**) clustering result showing three clusters and overlapped spikes.

For the raw data set, the spike detection technique employed in selective spike sorting identified 38,484 spike events and all the spike waveforms were extracted using a window of 31 samples with their peaks aligned at the 12th index, as shown in Fig. 10(a). Interpolation was employed to improve resolution of each spike waveform and appropriate attributes were extracted for clustering. Figure 10(b) shows three clusters and the overlapped spikes window, following a full exploitation of super paramagnetic clustering and feature selection. Figure 6(a) shows the putative spike shapes, estimated for each cluster using the simple regression method. The background noise was tailored to represent a coiflet type wavelet as shown in Fig. 6(c). The original data was optimised by stationary decomposition of the original data through the estimated wavelet. The spike shapes extracted using the decomposed original data are shown in Fig. 6(b).

**Figure 11:**
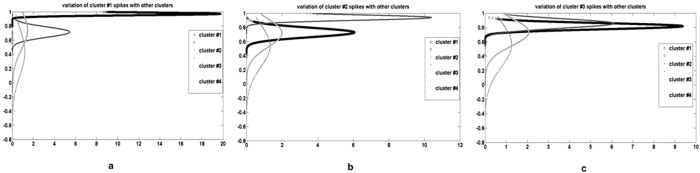
Cross correlation graph of putative spike shapes from each cluster with rest of spikes.

The prediction and elimination algorithm statistically incorporates the spike rates 0.0015, 0.0007 and 0.0001 estimated for each cluster, respectively, to individually target the correlated spike shapes. The effect of the likelihood principle is displayed in Fig. 11. Figure 11(a,b) display a strong similarity with spikes in their respective clustered groups while Fig. 11(c) depicts the effect of overlapping, where the spike shapes of clusters-1 and clusters-2 establishes a strong correlation with those of cluster-3.

**Figure 12:**
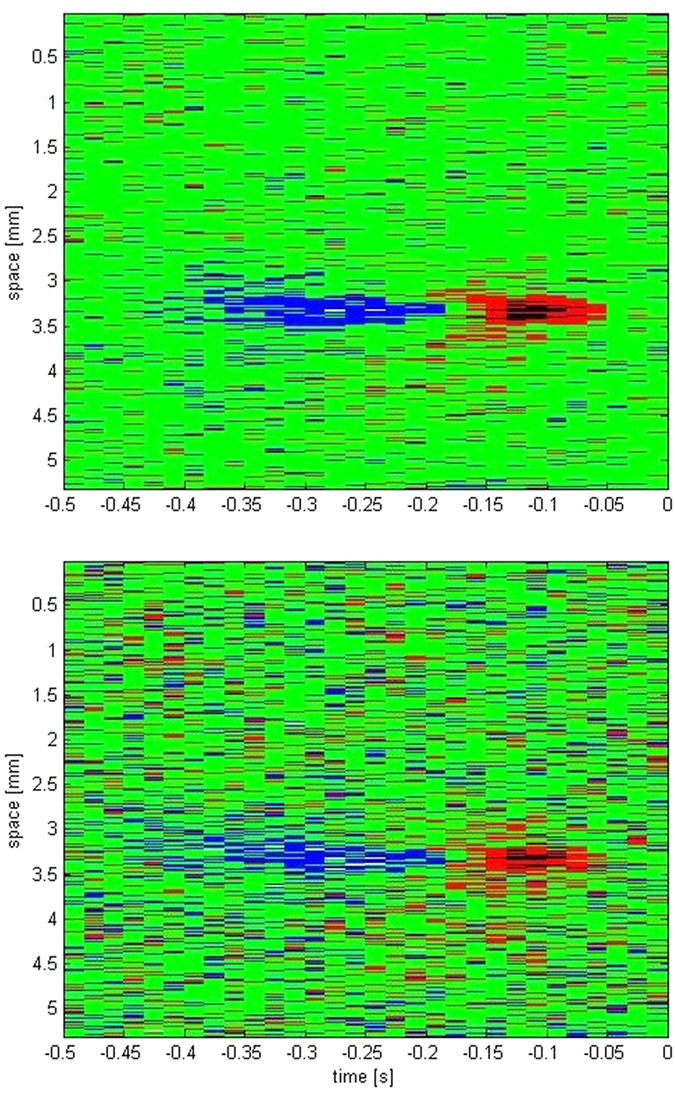
Comparison of stimuli response for low (top) and high spike counts (bottom).

The significance of the proposed algorithm can be realized through following analysis. We use the same procedure as in refs 3 and 38 which involves approximating the stimuli response of each cell to an evoked frame on the display, so as to locate the spatial and temporal response fields of the retina. Figure 12 shows two conditions of the recovered spatio-temporal stimulus response of cell 4. The image quality in Fig. 12 (top) is high as compared to that in Fig. 12 (bottom), owing to fact that Fig. 12 (Top) is constructed using 16000 stimuli responses while Fig. 12 (bottom) is constructed with just 4000 stimuli responses. The enhanced image quality in Fig. 12 (Top) was a result of algorithms capability to successfully identify and sort the overlapping and distorted spikes. This increase in true spikes to false positives ratio added extra pixels to the image, thereby receptive fields were more distinct.

One of important attributes of the algorithm is its ability to address correlated spike waveforms. A commonly accepted qualitative evaluation method in the absence of ground truth is by percentage similarity estimation i.e. coefficient of determination and another important technique is to employ correlation analysis^24^,^42^. To be adaptable for either of the analysis methods, we generate a pseudo temporal voltage information similar to synthetic data generation technique described in^36^ to compete with the original data. For each identified neuron, their individual voltage contribution 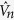 is estimated, in an effort that the sum of individual voltage’s 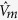 should resemble the original data *V*_*m*_. To do so, a temporal binary impulse response map *δ*(*n, t*) of each neurons spiking activity on an electrode is created. The estimated putative spike shape 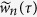 of each cluster on any channel as shown in 11*a* is fit into impulse area of temporal region such that


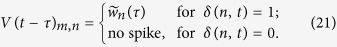


where *m* represents the channel identity or electrode number, *n* is the approximated neuron number and *t* is the time sample in integer. A channel voltage matrix at any spiking event is prepared to observe the correlation activity as shown in Fig. 10(a) for cell 4 and 10(b) for cell 5. As an example, Fig. 10 displays peak spikes on channel 28 for both cell 4 and cell 5 indicating a correlation activity. This information is in turn used to estimate their respective impulse response and eventually to calculate individual temporal spike train or voltage response 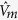 using 21.

**Figure 13:**
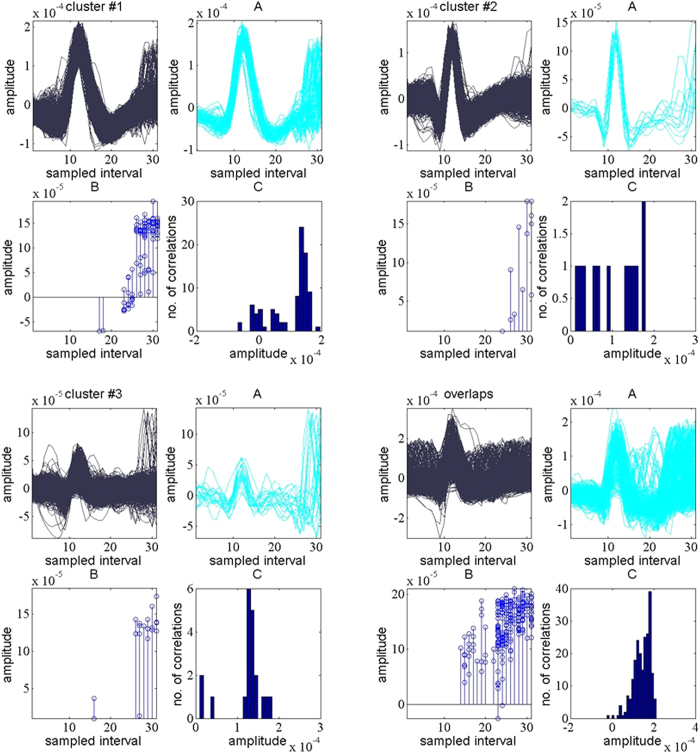
Correlated waveforms and correlation index results for each cluster from channel 28 (**A**). Correlated waveforms identified for respective clusters, (**B**). Time samples at which overlapping were identified, (**C**). Overlapping amplitudes resolved by the algorithm.

Comparing 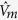 before and after processing the data *V*_*m*_ through the algorithm, correlation index is estimated for individual channels by identifying correlated channel and the respective neurons contributing to the channel. The results for the identified correlated channel 28 is shown in Fig. 13 where, identified correlation times to the right of peak in every cluster is shown in 13*B* and the resolved secondary amplitude is shown in 13*C*. Figure 13(A) also shows the algorithms ability to clearly isolate the correlated spike waveforms. Additionally, coefficient of determination was estimated for 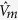 and channel data *V*_*m*_ by taking square of pearson’s correlation index. Ultimately, the coefficient of determination for correlated channels ranged between 95% to 98% similarity. The algorithm was capable of identifying additional 4682 spike events in channel 28.

## Discussion and Conclusion

The ground truth information provided by the synthetic data sets has been effectively exploited to construct an efficient spike sorting algorithm. With the assistance of the generative model it is possible to explore the unknown distribution of spikes, spike train, and background noise resulting in the introduction of many key improvements which have not been possible with unknown synthetic data. The results for the synthetic and raw data clearly indicates that SPC performs better in clustering the spikes and could be employed as a default approach for establishing the partial ground truth. The selective spike sorting algorithm is convincingly effective because dependency of the performance is equally distributed throughout the unified sequence of the unit. The algorithm does not demand full identification at every ramification but rather depends on accuracy of the output. This motivation has resulted in reduced false identification, greatly reflecting on the improved efficiency of clustering algorithms. These results establish a strong rationale for the prediction and elimination methodology to work through the raw voltage data and sort the identified spike events.

Another significant advantage of selective spike sorting is that it provides the flexibility to analyse every data channel individually, irrespective of MEA or tetrode based recording. The ambiguity in selecting the appropriate cluster procedure is eliminated by the flexibility of formatted clusters offered in this algorithm. We incorporate threshold techniques to identify the spike shapes, and normalise the data as a preliminary step to improve the performance of CoB. The performance of COB is depends on amplitude of the neuron and response of spikes for bispectrum. This is really important for spike detection as its functioning is independent of shape of spike and depends on the frequency of spiking interval. The novel feature selection process and the clustering procedure bolster the spike detection procedure, establishing sufficient ground truths, and does not have to just rely on probabilistic models. The statistical estimation method exploits the wavelet featured background noise to decompose any overlapping spikes and identifies the spikes on the basis of its shape and, spike rate at any pre-established spike event. Additionally, the inter spike interval sets a quantitative threshold on the length of spike shape; therefore any secondary spikes from the same cluster appearing inside the interval are neglected. With SPC, it is also possible to isolate uncharacteristic overlapping spikes which pose a problem with Klustakwik.

The performance of selective spike sorting is not limited to processing of individual data channel. The extracellular data using the tetrode electrode, also provides intracellular spike shapes to match the spike events^43^, which could be used as references in designing the spatio-temporal filter. Multiple channel information, as in tetrode electrodes where the number of channels is fewer than that in an MEA, it is possible to tailor the noise covariance filter to be effective both in space and time. The real time realization of the algorithm combined with wireless *in vivo* monitoring techniques^44^ will lead to a state of the art system. This would be a remarkable achievement and open up neurophysiological studies to whole new exploring environment.

## Additional Information

**How to cite this article**: Veerabhadrappa, R. *et al*. Unified selective sorting approach to analyse multi-electrode extracellular data. *Sci. Rep.* **6**, 28533; doi: 10.1038/srep28533 (2016).

## Figures and Tables

**Table 1:** Comparison of clustering results formed by each of the ad hoc clustering algorithms.

Data set	Ground Truth with number of clusters and spikes in each cluster	Wave_clus	Klustakwik	OPTICS	Selective sorting (SPC)
D1n-0.05	1115	792	491	977	1010
	1113	1025	516	881	914
	1155	531	659	946	943
		494	343	393	113*
		217*	712		
			48		
			50		
D1n-0.1	1164	639	400	2982	1000
	1155	470	633	126	981
	1129	653	45	15	971
		424	1072	13	92*
		1059*	609	139	
			435		
D1n-0.15	1159	1054	420	2939	909
	1172	720	529	279	856
	1141	675	491		803
		360	1087		162*
		409*	633		
			15		
D1n-0.2	1136	897	483	2010	962
	1099	784	426	221	841
	1179	439	404		909
		111*	419		135*
			126		
			72		

The values in the table indicate the number of clusters estimated and spikes allocated to each cluster. *Indicates the cluster that constitutes overlapping spikes. First column presents the ground truth information about the synthetic data sets used with actual number of clusters and number of spikes in each cluster.

**Table 2:** A comparison of final results obtained by unified continuous basis pursuit and unified selective sorting after successfully sorting the overlapping spikes.

Data set	Greedy pursuit (Continuous basis pursuit)	Missed spikes	False positives	Selective Sorting	Missed spikes	False positives
	Sorted			Sorted		
D1n01	3300	148	58	3291	157	5
D1n02	3373	41	125	3284	130	1
D1n005	3223	160	44	3249	134	2
D1n015	3376	96	92	3365	107	1
